# American Dental Association

**Published:** 1883-08

**Authors:** 


### AMERICAN DENTAL ASSOCIATION.

The coming meeting of this, the representative American Society, promises to be of more than usual interest. Niagara is the favored place of meeting, and the sessions held there are always successful. The first preliminary meeting, which resulted in the

American Dental Association, was held there in 1859, and since then of the twenty-one annual meetings, six have met there, so that about every third meeting is held at Niagara. There are many reasons for this choice, but there is one which is quite sufficient without considering others, and that is that the sessions held there are never failures.

The Association meets this year under the shadow of a heavy affliction. Its venerable and beloved President, Dr. W. H. Goddard, who had so long and so faithfully served as its treasurer, has died during his term of office. There is no one who was present at his assumption of the gavel at Cincinnati last year, and who marked his honest exultation in that culmination of a long and honorable professional life, who will not deeply regret the loss, not alone for society reasons, but on Dr. Goddard's own account, that he was not permitted the gratification of presiding at the meetings of a society whose interests had so long laid near his heart. Surely, it will be a source of much pleasure to every member to know that he had the satisfaction of standing the representative head of the profession in America before he died.

The sessions will necessarily be conducted by Dr. G. J. Friedricks, of New Orleans, the First Vice President, and there is no reason to doubt that he will be fully equal to the occasion.

